# DNA Methylation in Circulating Tumour DNA as a Biomarker for Cancer

**Published:** 2008-01-25

**Authors:** Ruth E Board, Lucy Knight, Alastair Greystoke, Fiona H Blackhall, Andrew Hughes, Caroline Dive, Malcolm Ranson

**Affiliations:** 1 Clinical and Experimental Pharmacology, Paterson Institute of Cancer Research, Wilmslow Road, Manchester M20 4BX; 2 Discovery Medicine, AstraZeneca Pharmaceuticals, Alderley Park, Maccles-field, Cheshire. SK10 4TG; 3 CRUK Department of Medical Oncology, Christie Hospital NHS Trust, Wilmslow Road, Manchester M20 4BX; 4 Professor, Discovery Medicine, AstraZeneca Pharmaceuticals, Alderley Park, Macclesfield, Cheshire. SK10 4TG; 5 Professor, Clinical and Experimental Pharmacology, Paterson Institute of Cancer Research, Wilmslow Road, Manchester M20 4BX; 6 Professor, CRUK Department of Medical Oncology, Christie Hospital NHS Trust, Wilmslow Road, Manchester M20 4BX

**Keywords:** DNA methylation, biomarker, circulating tumour DNA, MSP, MethyLight

## Abstract

Free circulating DNA, which is thought to be derived from the primary tumour, can be detected in the blood of patients with cancer. Detection of genetic and epigenetic alteration in this tumour DNA offers a potential source of development of prognostic and predictive biomarkers for cancer. One such change is DNA methylation of the promotor region of tumour suppressor genes. This causes down regulation of tumour suppressor gene expression, a frequent event in carcinogenesis. Hypermethylation of the promotor region of a number of genes has been detected in many tumour types and more recently these changes have been detected in circulating tumour DNA. This review will summarise the literature detailing DNA methylation in circulating tumour DNA and discuss some of the current controversies and technical challenges facing its use as a potential biomarker for cancer.

## Introduction

There is currently great interest in the development of tumour biomarkers to aid the management of cancer. “A biomarker is a characteristic that is objectively measured and evaluated as an indicator of normal biologic processes, pathogenic processes, or pharmacologic responses to a therapeutic intervention” (National Institute of Health Biomarkers working party, 2001).

Tumour biomarkers in current clinical use are generally proteins secreted by the tumour such as CA-125 (Rustin et al. 1996) and PSA (prostate specific antigen) (Kelly et al. 1993), or cell surface receptors that can predict response to specific targeted treatments, such as HER2 overexpression in breast cancer which is predictive of response to targeted inhibition of HER2 with trastuzumab (Baselga et al. 1996). However, similar tumour markers are not available for all cancers and many of the available markers have shortcomings in terms of sensitivity and specificity.

The development of robust blood-based biomarkers that accurately reflect the host tumour is an emerging field. Whilst DNA alterations can be assessed within a primary tumour sample, a less invasive and ‘patient friendly’ option is detection of these changes within patients’ blood. Biomarkers which require a tumour sample can impose constraints on clinical utility particularly in disease settings where obtaining tumour tissue prior to treatment is problematic (e.g. pancreatic, CNS tumours). Often tumours are inaccessible to biopsy and repeated biopsies of a tumour to assess changes in biomarkers are rarely practical or acceptable to patients. The correlation of alterations in tumour DNA with circulating tumour DNA would allow potential development of clinically relevant biomarker blood tests for early detection of cancer, prediction of a likely treatment effect and assessment of tumour response to therapy.

DNA-based biomarkers are an attractive option compared to RNA and protein-based biomarkers. DNA is inherently stable compared to RNA which is rapidly degraded at room temperature. Thus it is more feasible to isolate and analyse DNA and extraction can be performed on stored and archival samples.

Advances in molecular biology have lead to a rapid growth in the understanding of genetic and epigenetic change in cancer. Modern molecular detection methods such as polymerase chain reaction (PCR)-based assays can detect and quantify small amounts of nucleic acids and these technologies offer a potentially low cost, high throughput and sensitive method for the evaluation of genetic change in cancer. Recently the role of methylation in altering gene expression has been described and this has been detected in both tumour and circulating tumour DNA. This review will discuss some of the technical challenges related to the study of circulating tumour DNA, outline the role of DNA methylation in cancer and summarise the current evidence supporting the potential use of DNA methylation in circulating tumour DNA as a potential biomarker for cancer.

## Circulating Tumour DNA

Small amounts of free DNA (approximately 1ng of soluble DNA/ml) are found circulating in the plasma of healthy individuals, (Shapiro et al. 1983). Increased levels have been reported in the serum or plasma of patients with a variety of illnesses including autoimmune disease, chronic inflammation and cancer (Shapiro et al. 1983; Koffler et al. 1973; Leon et al. 1981). Increased levels of circulating DNA have also been described after exhaustive exercise or trauma (Atamaniuk et al. 2004; Lo et al. 2000). The serum of cancer patients is enriched in DNA containing on average around 4 times the amount of DNA compared to normal controls (Shapiro et al. 1983), although this is variable and overlap exists between the two groups. Initial studies demonstrated that higher levels of circulating DNA could be detected in the serum of patients with metastatic disease compared to those patients with localised disease (Leon et al. 1977) and more recently it has been estimated that in Dukes stage B colorectal cancer 0.15% of tumour DNA is fed into the circulation each day increasing to over 3% in more advanced disease (Diehl et al. 2005). Further studies have demonstrated a correlation between circulating DNA levels and prognosis in a number of tumour types (Maebo, 1990).

The mechanism and source of DNA release remains largely uncertain and a number of potential explanations have been explored including release of DNA from either malignant or haemopoietic apoptotic cells and release of DNA from necrotic cells.

One hypothesis is that DNA is released due to lysis of circulating cancer cells or micrometastases. It is well established that metastatic tumour cells can circulate in the blood however circulating DNA can be detected in the plasma of patients in whom circulating metastatic cells are not found (Castells et al. 1999) and where there may not be enough circulating cells to justify the amount of DNA detected (Anker et al. 1999).

DNA released from apoptotic cells is uniformly digested into 185–200bp fragments (equivalent to whole-number multiples (1–5x) of nucleosomal DNA). DNA with fragment sizes typical for apoptosis has been demonstrated in the plasma of patients with systemic lupus erythrematosus and in patients following haemodialysis (Atamaniuk et al. 2006). It is suggested that the majority of this DNA is released from apoptotic leucocytes. In support of this, sex mismatched bone marrow transplantation patients have been used to demonstrate that the majority of circulating DNA in these patients comes from the haemopoietic system (Lui et al. 2002). The contribution of DNA from the haemopoetic system in patients with epithelial tumours is yet to be determined. One study found little contribution of T-cells to the bulk of plasma DNA in patients with solid tumours (Jahr et al. 2001).

The source of the DNA in cancer patients remains an ongoing area of investigation. It was not until the 1990s following refinement of PCR-based technologies that the neoplastic characteristics of plasma DNA in cancer patients were recognised. In 1994 Sorensen and coworkers demonstrated that mutated K-Ras sequences could be detected by PCR in the plasma or serum of patients with pancreatic cancer (Sorenson et al. 1994) and in the same year point mutations in the N-Ras gene were documented in the plasma of patients with myelodysplastic syndrome (Vasioukhin et al. 1994). More recently it has been shown that tumour specific epigenetic alterations such as hypermethylation of promoter region of tumour suppressor genes can be detected in the plasma DNA of cancer patients (see Table 1 for summary) confirming that, at least in part, circulating DNA is derived from the primary tumour. The proportion of circulating DNA that is tumour-derived varies amongst studies from less than 3% to more than 90% (Jahr et al. 2001) and from 0.02% to 8–11% (Diehl et al. 2005). It is not yet known whether these differences result from the different methodologies used or whether they reflect distinct biological behaviour in different tumours types.

It has been proposed that as tumours enlarge there is hypoxia-induced necrosis of both the tumour cells and surrounding stroma and inflammatory cells (Jahr et al. 2001). These cells may be phagocytosed with subsequent release of DNA into the circulation containing both tumour specific and wild type sequences. Diehl et al. 2005 demonstrated that the majority of mutated APC sequences in patients with colorectal cancer were detected in the smaller size fragments of circulating DNA, whereas larger fragments tended to be wild-type.

Jahr and co-workers observed an increase in total DNA concentration following either apoptosis (CD95) or necrosis (acetaminophen) induced liver damage in mice. DNA of a higher molecular weight was observed following induction of necrosis alone (Jahr et al. 2001). This suggests that the DNA in cancer patients comes from both an apoptotic and necrotic source and that the higher molecular weight fragments originate from cell necrosis. In contrast to DNA extracted from healthy individuals, DNA from cancer patients has a different pattern of fragment sizes. It has been demonstrated that the integrity of DNA extracted from serum or plasma is significantly increased in patients with colorectal, gynaecological and breast cancer compared to normal controls (Umetani et al. 2006; Wang et al. 2003). Further studies are planned to investigate whether elevated levels of longer DNA fragments in blood may provide a useful biomarker in the management of cancer.

The clearance mechanism of circulating DNA is not yet known and it is not clear whether differences exist between healthy individuals and cancer patients. It has been demonstrated that patients with stomach and colorectal cancer have lower levels of DNAse activity compared with controls (Skvortsova et al. 2006). The half-life of DNA extracted from haemodialysis patients was determined to be approximately 4 minutes (Rumore et al. 1992) and the half life of circulating foetal DNA in the maternal system is 16 minutes (Lo et al. 1999). To date no studies have investigated the half-life of circulating DNA in cancer patients. The role of hepatic and renal clearance has also not been investigated in detail although it has been demonstrated that mutated DNA sequences can be detected in the urine of colorectal and pancreatic cancer patients suggesting renal excretion as one potential clearance mechanism (Su et al. 2004).

Despite the uncertainties surrounding the precise source and role of circulating DNA, many studies have now demonstrated the presence of tumour-specific changes in circulating DNA providing the opportunity to develop blood borne biomarkers for cancer. One such change is the methylation of the promoter regions of tumour suppressor genes.

### DNA methylation

Classically, cancer has been thought of as a disease driven by genetic abnormalities including mutations in tumour suppressor genes and oncogenes. However, more recently it has become apparent that epigenetic alterations also play a role in cancer progression and its maintenance. The term “epigenetic” refers to altered patterns of gene expression mediated by changes other than an alteration in the primary nucleotide sequence. One such epigenetic change is abnormal DNA methylation. In the mammalian genome methylation takes place primarily at cytosine bases. Methylation is catalyzed by DNA methyltransferase enzymes that use s-adenosyl-methionine as a methyl donor to replace a hydrogen atom with a methyl group at the carbon 5 position of the cytosine pyrimidine ring (Ramsahoye et al. 2000). This only occurs at cytosine bases located 5′ to a guanosine in a CpG dinucleotide (Herman and Baylin, 2003). Methylation of CpG sites within promoter regions is an important mechanism for controlling gene expression in normal cells (Klose and Bird, 2006). There are three main mechanisms which link methylation to cancer: transcriptional silencing *via* hypermethylation in the promoter regions of tumour suppressor genes, hypomethylation (demethylation) resulting in the failure to repress expression of tissue restricted or proto-oncogenes and genome wide hypomethylation leading to increased mutation rates and chromosome instability (Feinberg et al. 2002). CpG dinucleotides occur throughout the genome but are clustered in short regions of 0.5–4kb in length, known as CpG islands (Bird, 2002). Most CpG islands are located at the promoter regions of genes and are unmethylated in normal cells. Hypermethylation of these regions causes altered transcription or “silencing” of the target gene. This may be a natural event, for example fully methylated islands are associated with many silent genes on the inactive X chromosome in females. However, it is now recognised that the epigenetic programme of human tumours is frequently abnormal leading to adverse transcriptional profiles and increased genetic instability (Robertson, 2005). Loss of methylation may affect the stability of chromosomal structure as demonstrated among patients with the rare immunodeficiency centromeric instability and facial abnormalities syndrome (ICF, MIM#242860). These patients have failure to thrive, recurrent infection and variable mental retardation due to reduced levels of DNA methyltransferase 3B, which results in loss of methylation at selected centromeric regions and marked alterations in chromosomal structure (Esteller et al. 2000).

Epigenetic modification may be a more frequent cause of loss of function than genetic defects (Jones and Baylin, 2002). Promotor hypermethylation has been observed at well characterised tumour suppressor gene sites that can cause cancer when mutated in the germ line such as BRCA1 (Esteller et al. 2000) and VHL (Herman et al. 1994). A number of candidate tumour suppressor genes have been identified on the basis of promoter hypermethylation in different cancer types (Esteller, 2007). Promoter methylation may be the only cause of the reduced expression of these genes in cancer with mutations less frequently observed.

### Detection of DNA methylation

Three different needs in the field of methylation analysis have been identified; genome wide estimation of DNA methylation, quantitative methylation measurements (detection down to 5% methylated DNA) and ultra sensitive quantification (detection down to ≤ 1% methylated DNA). A number of reviews have been published detailing the plethora of methods available (Laird, 2003; Brena et al. 2006) but, as methylation analysis is an emerging field, new detection methods are regularly released to the research community. With this in mind a detailed description of all available methodologies is outside the scope of this review. Briefly, the majority of methods require three main steps:

1. An initial step to differentiate between methylated and non methylated DNA in order to make this difference analyzable. Methods most commonly used are:
  - Bisulfite conversion of genomic DNA (Frommer et al. 1992).
    Here, methylation is not retained during the amplification process of PCR, a step which is required by several methylation assays. Treatment of DNA with sodium bisulfite allows conversion of genomic DNA, retaining the methylation pattern. Unmethylated cytosines are deaminated to uracil by sodium bisulfite and methylated cytosines are resistant to modification and remain as cytosine
  - Physical separation using DNA binding proteins with high specificity for methylated DNA or
  - Endonuclease digestion
    Here, isoschizomers of bacterial restriction enzymes with different sensitivities for 5-methylcytosine will preferentially cut either methylated or non-methylated DNA. However, this is limited by the fact that this will only inform on the methylation status of the cytosine residues recognized by the methylation-sensitive restriction enzymes used.
2. Many analysis applications require an amplification step, usually by PCR, to enable methylation detection. There are a number of options depending on primer design. In simple terms the PCR can be methylation specific (MSP) or independent.
  MSP, the most widely used method of DNA methylation analysis, uses primers that bind specifically to methylated or non-methylated DNA. This is an ultra sensitive method of detection commonly used for the detection of very few molecules of methylated DNA in an excess of non-methylated DNA (sensitivity reported as 1:1000) (Herman et al. 1996).
  Methylation independent PCR is achieved by excluding any CpG site form the primer hybridization sites, commonly used for the quantitative detection of DNA methylation in the range of 5% to 95% percent methylated DNA. Whilst not as sensitive as MSP, this method is useful for the quantitative or detailed analysis of 5-methylcytosine distribution.
3. Following amplification a number of detection methods are available to analyze the results. The most common include capillary sequencing, pyrosequencing, mass spectrometry, gel electrophoresis and RT-PCR.

Many publications assessing methylation in patients with cancer have used conventional MSP amplification with gel electrophoresis readout. This is an ultra sensitive method of analysis and due to its simplicity it is widely used in its field. However, MSP detects only the presence or absence of methylated alleles in a “yes or no” fashion and is not quantitative. Furthermore, unless DNA is also amplified with wild type primers specific for unmodified DNA, there is no control for adequacy of bisulphite treatment which may lead to false positives if non methylated cytosines have been inadequately converted to uracil. There is also potential for mis-priming especially with high numbers of PCR cycles (Shaw et al. 2006).

A large number of techniques are available for quantitative analysis (Brena et al. 2006) including bisulfite sequencing from clones, pyrosequencing (Tost et al. 2003; Colella et al. 2003) or mass spectrometry in combination with methylation independent PCR as well as a number of quantitative PCR methods based on the detection of methylated sequences via fluorescence in the PCR reaction, e.g. MethyLight (Eads et al. 2000), HeavyMethyl and quantitative analysis of methylated alleles (QAMA) (Zeschnigk et al. 2004). These have individual advantages and disadvantages, mainly related to the concentration of DNA required for analysis and the sensitivity of the test.

Many of the techniques described above are suitable for analysis of DNA methylation in a specific gene promoter. A number of assays are also available for the measurement of genome wide methylation using techniques such as high-performance liquid chromatography (HPLC) or high performance capillary electrophoresis (HPCE). It is also possible to detect DNA methylation in discrete areas of the genome such as CpG islands with restriction landmark genomic scanning (RLGS) or bacterial artificial chromosome (BAC) arrays. Furthermore, novel technologies that make use of CpG island and promoter micro-arrays can efficiently study methylation at a genome wide level. These techniques are less suitable for development of biomarkers due to the large quantities of DNA required, but they have been successfully employed to monitor global changes in methylation and to discover novel tumour suppressor genes.

It should be emphasized that generally no single technique or general approach is superior to the others. The method of choice will depend on the desired application and the goals of the study planned.

### Hypermethylation in plasma and serum

Promotor methylation has been reported in a number of different genes across a range of tumour types (Rodenhiser and Mann, 2006). Some genes are methylated in a number of cancers whereas others appear more specific for certain tumour types. The first studies to show it was possible to detect methylation changes in serum DNA from cancer patients were reported by Esteller and co-workers (Esteller et al. 1999) in 1999 in NSCLC patients and by Wong and co-workers (Wong et al. 1999) who demonstrated p16 methylation in plasma and serum of patients with liver cancer. Subsequently a number of studies have evaluated the potential of circulating tumour DNA methylation in serum and plasma for the molecular diagnosis and/or prognosis of a variety of cancer types. The most extensively studied are summarised in the table below.

In general there is concordance between the methylation changes in plasma or serum and those changes in the primary tumour. Most often if DNA methylation is detected in the serum or plasma then the primary tumour will also be positive for the same alteration (Skvortsova et al. 2006; Esteller et al. 1999; Wong et al. 1999; Bearzatto et al. 2002; Ramirez et al. 2003; Zou et al. 2002; Fiegl et al. 2005; Dulaimi et al. 2004). However, exceptions to this have been reported in small numbers of cases (Fujiwara et al. 2005). The reasons for the presence of methylation in the blood when it is absent in the primary tumour are not clear. This may simply be due to a heterogenous clonal malignant population such that arbitrary tumour sampling failed to biopsy regions of methylation.

### DNA methylation in normal tissue

An important measure of an effective biomarker assay is the sensitivity and specificity of the test. Sensitivity refers to the proportion of cases with disease that test positive i.e. true positives, and specificity refers to the number of control cases (people without disease) who test negative i.e. true negatives. If methylation markers can be identified in tumours which are absent in normal cells then this may yield an important potential screening tool. DNA methylation changes have been reported to occur early in the carcinogenic process and are potentially good indicators of early disease (Laird, 2003). Molecular analysis on tumour adjacent ‘normal tissue’ has detected a number of genetic alterations including methylation. (Hoque et al. 2006; Shen et al. 2005). The reasons for methylation in the apparent absence of disease are as yet not well defined but there may be a number of explanations.

1. These alterations may be explained by the concept of field cancerization (or field defect); a term originally used to describe the discovery of histologically abnormal epithelium surrounding oral cancers (Slaughter et al. 1953). As it has become increasingly established that an accumulation of genetic alterations takes place transforming a normal cell to a cancer cell (multi-step carcinogenesis, (Fearon and Vogelstein, 1990)) a molecular basis for field cancerization can be described. It is hypothesised that a stem cell acquires a genetic alteration and forms a patch with identical daughter cells. The lesion gradually becomes a field, laterally displacing the normal epithelium. As the lesion becomes larger additional genetic hits give rise to various subclones, one (or more) of which may develop into an invasive cancer (Braakhuis et al. 2003). Hence histologically ‘normal’ epithelium adjacent to a cancer may harbour cancer associated genetic changes.
2. Methylation may be a consequence of environmental exposure to a carcinogen such as tobacco or another as yet unidentified compound. DNA methylation changes are more common in prostate and lung cancer tissue of smokers (Enokida et al. 2006; Liu et al. 2006). In a study of lung cancer survivors, never smokers and cancer free smokers, DNA methylation was more common in smokers than in non smokers (Belinsky et al. 2005).
3. Methylation has been shown to increase with increasing age in colorectal mucosa (Issa et al. 1994; Ahuja et al. 1998). Extensive evaluation of methylation changes in normal tissue needs to be undertaken before one can be certain of the specificity of any assays.
  Whilst DNA methylation in normal tissue may make discriminatory assays less sensitive, in those studies of circulating DNA that include disease free populations as control cases, the specificity of DNA methylation has been remarkably high (Bearzatto et al. 2002; Usadel et al. 2002; Zou et al. 2002; Dulaimi et al. 2004; Wong et al. 2000; Lee et al. 2002; Goessl et al. 2000). In these trials, promotor methylation of the target genes has only been detected in the serum or plasma of those patients with cancer and not control subjects. However, more recently in a study reported by Hoque and coworkers (Hoque et al. 2006), the frequency of methylation of four genes (APC, GSTP1, RASSF1A and RARβ2) was determined in the plasma of breast cancer patients using quantitative MSP. Methylation of at least one gene resulted in a sensitivity of 62% and a specificity of 87%. Methylation was detected in the plasma of 13% of control subjects which may be explained by the more sensitive detection methods used in this study.

### DNA methylation as a prognostic and predictive biomarker

DNA methylation has been investigated as a potential prognostic and predictive biomarker (Table 2). The presence of tumour specific methylation markers in the serum or plasma has been reported to be of prognostic significance in a number of tumour types which supports a role for methylation in cancer progression.

### Other types of clinical sample for extraction of DNA

DNA methylation has been detected in a number of bodily fluids of patients with cancer, for example urine, saliva and ascitic fluid. In some tumour types this may be an alternative to tissue biopsy and like blood samples may provide a less invasive approach to the monitoring of biomarkers compared to tissue sampling. Cancers of the bladder, kidney and prostate contribute cellular DNA to urinary sediment (Perry et al. 2006) and this DNA can be analysed for panels of methylation markers. Promotor methylation of the GSTP1 gene has been detected in the urine and ejaculates of men with prostate cancer but not in patients with benign prostatic hyperplasia (Goessl et al. 2000). A combination of four genes (p16, ARF, MGMT, and GSTP1) has been proposed to detect prostate cancer with an estimated 87% sensitivity and 100% specificity (Hoque et al. 2005).

In lung cancer, DNA methylation changes can be detected in the sputum of patients (Machida et al. 2006). It has been suggested that this may also play a role in early detection and assessment of lung cancer risk. Methylation of the p16 and MGMT promotor regions has been detected in the sputum up to 3 years prior to the diagnosis of squamous cell carcinoma (Palmisano et al. 2000) and more recently panels of genes have been evaluated as possible markers for early lung cancer detection with a sensitivity and specificity of 65% (Belinsky et al. 2006).

Methylated genes have been detected in peritoneal and ascitic fluid of patients with ovarian cancer (Ibanez de Caceres et al. 2004; Muller et al. 2004). One study demonstrated that methylation was specific for patients with cancer with no methylation detected in any of the genes in peritoneal fluid from patients with benign ovarian disease (Ibanez de Caceres et al. 2004).

In breast cancer, methylation of genes can be detected in cancer cells collected by ductal lavage (Evron et al. 2001) and in nipple aspirate fluid (Krassenstein et al. 2004). And, in head and neck cancers, methylation of p16, DAPK and MGMT have been detected in saliva (Rosas et al. 2001).

### Development of a panel of markers

In order to increase the sensitivity and specificity of genetic and epigenetic biomarkers many combinations and panels of genes have been investigated to see if a combination of markers will improve sensitivity and specificity of assays. Many studies combine a number of methylated genes to try and identify a panel of markers which are more specific than a single change on its own. New technologies such as gene arrays have been adapted to screen hundreds of promotor regions to yield a more specific “signature” or methylation phenotype for cancer versus normal tissue. Multiple methylation events often termed the CpG island methylator phenotype (CIMP) have been described in a number of cancers, most extensively in colorectal cancers.

Colorectal cancer is associated with a number of genetic and epigenetic alterations. One of the most important is the presence of multiple micro-satellite repeats, short repetitive DNA sequence tracts, termed microsatellite instability (MSI). MSI is also found in about 15% of sporadic CRCs and is usually the result of acquired loss of the mismatch repair gene hMLH1 (Thibodeau et al. 1993). Studies of methylation in colon cancer revealed increased frequencies of promoter methylation in the mismatch repair gene MLH1 in MSI-positive tumours suggesting that hypermethylation leads to MSI through silencing of MLH1. Subsequently a number of studies have added to the list of genes preferentially hypermethylated in sporadic MSI-positive cases of colorectal cancer including p16, TIMP and MINTS –1, –2 and –31 (Whitehall et al. 2002; Hawkins et al. 2002; Toyota et al. 2000). Further research has demonstrated that CIMP positive cancers represent a unique subset of tumours. They are more common in older patients, proximal colon cancers and in women and are also associated with distinct genetic alterations and high mutation rates in the KRAS and BRAF genes (Issa, 2004).

Similar distinct subsets of cancer based on tumour hypermethylation have been described (Esteller et al. 2001) in a number of other tumours including ovarian (Wei et al. 2006), gastric (Toyota et al. 1999) and pancreatic cancers (Ueki et al. 2000). Further study will determine which panels of genes are most effective as biomarkers for population screening, prediction of response to therapy and prognosis. Much more work is required on methylation detection in circulating DNA to develop these tests into clinically applicable resources.

The different methylation patterns detected in the same histologically defined group of cancers may indicated important differences in tumorigenesis and may help identify subsets of patients which differing molecular characteristics in the tumour which confer a particular clinical behaviour. Methylation status analysis may further both our understanding of the pathogenesis of cancer and may provide useful tools in the move to “personalised” treatment for cancer based on particular molecular change.

## Conclusions

Increasing knowledge of epigenetic alterations in cancer has provided unprecedented opportunity to evaluate the use of these changes as potential biomarkers in cancer. It is becoming increasingly apparent that these alterations can be detected in the blood of patients with cancer, highlighting potential for application in routine clinical practice. Already a number of potential markers or panels of markers have been identified in the plasma of patients with a number of cancers. If aberrant DNA methylation can be detected in the plasma this could have a potential use as an early indicator of disease in an asymptommatic population or as a predictive or prognostic test in the case of known disease. However, there are several caveats to the use of blood-borne biomarkers as surrogates for tumour biomarkers. Whilst positive plasma or serum assays are often only found in those patients with positive assays in the primary tumour, a proportion of patients will not have detectable alterations in the blood despite harbouring changes in the primary tumour. Many questions remain unanswered. Is a negative result is due to a real absence of methylated sequences?. Or are such sequences present, but at a level not detectable by current tests? Is the presence or absence of methylated sequences related to the size or vascularity of the primary tumour? Have these sequences been already cleared from the circulation?

With continued improvements in technology and increasing sensitivity of detection methods our understanding of the precise role of many of these changes will become clearer. Further work is required to refine, standardise and validate the extraction of DNA from plasma and the analytical methods use to measure and quantify genetic change. The incorporation of the study of biomarkers alongside traditional phase I, II and III clinical trials will further enhance our knowledge of the relevance of these changes to help guide treatment and target those patients most likely to benefit from specific therapies.

## Figures and Tables

**Table 1:** Methylation Markers in Circulating DNA from Cancer Patients.

Tumour type	Sample size	Methylation Markers	Tumour sample positive	Serum or Plasma positive No (%)	Method	Reference
NSCLC	22	p16 DAKP GSTP1 MGMT	9 (41) 5 (23) 2 (9) 6 (27)	3 (14) 4 (18) 1 (5) 4 (18)	MSP	(Esteller et al. 1999)
NSCLC	91	MGMT p16 RASSF1A DAPK RARβ2	ND	17 (18) 14 (15) 11 (12) 10 (11) 6 (7)	MSP	(Fujiwara et al. 2005)
NSCLC	35	p16	22 (63)	12 (34)	MSP	(Bearzatto et al. 2002)
NSCLC	50	RASSF1A DAPK TMS1	17 (34) 23 (45) 18 (35)	17 (34) 20 (40) 17 (34)	MSP	(Ramirez et al. 2003)
NSCLC	89	APC	95 (96)	42 (47)	MSP	(Usadel et al. 2002)
NSCLC	105	p16	73 (79)	77 (73)	MSP	(An et al. 2002)
NSCLC	115	14-3-3σ	ND	39 (34)	MSP	(Ramirez et al. 2005)
NSCLC	50	p16	(84)	(72)	MSP	(Liu et al. 2003)
NSCLC	61	p16 CDH13	(79) (66)	(26) (23)	MSP	(Ulivi et al. 2006)
CRC	58	p16	31 (53)	21 (40)	MSP	(Lecomte et al. 2002)
CRC	49	APC HMLH1 HLTF	nd	28 (57) in at least one marker	MethyLight	(Leung et al. 2005)
CRC	122	DAPK	67 (55)	3/14 (21)	MSP	(Yamaguchi et al. 2003)
CRC	52	p16	20 (38)	14/20 (70)	MSP	(Zou et al. 2002)
CRC	94	p16	44 (47)	13/44 (30)	MSP	(Nakayama et al. 2002)
Breast	148	RASSF1A	ND	29 (19) pre-treatment 33 (22.3) at 1 year	MethyLight	(Fiegl et al. 2005)
Breast	20	RASSF1A RARβ2	ND	3 (15) 3 (15)	MSP	(Skvortsova et al. 2006)
Breast	35	p16	8 (23)	5/8	PCR + restriction enzyme digest	(Silva et al. 1999)
Breast	34	RASSF1A APC DAPK	22 (65) 15 (44) 17 (50)	19 (56) 10 (29) 12 (35)	MSP	(Dulaimi et al. 2004)
Ovary	50	BRCA1 RASSF1A APC P14 P16 DAPK	12 (24) 25 (50) 5 (100) 3 (100) 5 (100) 3 (100)	7/43 (16) 12/43 (28) 4/5 (80) 1/3 (33) 4/4 (100) 2/3 (67)	MSP	(Ibanez de Caceres et al. 2004)
HCC	22	p16	16 (73)	13/16	MSP	(Wong et al. 1999)
HCC	25	p15	16 (64)	4/16 (25%)	MSP	(Wong et al. 2000)
HCC	29	p16	ND	23 (80%)	MethyLight	(Wong et al. 2003)
Renal cell cancer	26	APC ARF CDH1 GSTP1 MGMT P16 RAR-B2 RASSF1A TIMP3	5/17 (29) 4/17 (25) 10/17(59) 2/17 (12) 1/17 (6) 6/17 (35) 9/17 (53) 15/17 (88) 12/17 (71)	1/18 (6) 1/18 (6) 6/18 (33) 1/18 (6) 0/18 (0) 4/18 (22) 1/18 (6) 2/18 (11) 3/18 (17)	Quantitative real time PCR	(Hoque et al. 2004)
Head and neck	95	P16 MGMT DAPK GSTP1	26 (27) 31 (33) 17 (18) 0/41 (0)	8/26 (31) 14/29 (48) 3/17 (18)	MSP	(Sanchez-Cespedes et al. 2000)
Gastric	54	E-cadherin P16 P15 DAPK GSTP1	41 (75) 36 (67) 37 (68) 38 (70) 10 (18)	31/41 (57) 28/36 (52) 30/37 (56) 26/38 (48) 8/10 (56)	MSP	(Lee et al. 2002)
Oesophageal	52	APC	48 (92)	13 (25)	MSP	(Kawakami et al. 2000)
Bladder	27	p14 p16	15 (56) 5 (18)	13/15 2/5	MSP	(Dominguez et al. 2002)
Melanoma	50	RASSF1A RAR β2 MGMT	9/18 (50) 11/18(61) 2/18 (11)	13 (26) 10 (20) 5 (10)	MSP	(Mori et al. 2005)
Melanoma	107/109	ER-alpha	55/107 (51)	26/109 (24)	MSP	(Mori et al. 2006)
Prostate	33	GSTP1	16/17 (90)	23/32 (72)	MSP	(Goessl et al. 2000)
Prostate	85 (localised disease) 18 (HRPCa)	GSTP1	ND	10 (12) 5 (28)	Restriction endonuclease PCR	(Bastian et al. 2005)

**Abbreviations:** CRC, colorectal cancer; NSCLC, non small cell lung cancer; HCC, hepatocellular cancer; RASSF1A, Ras-association domain family 1A; GSTP1, glutathione S-transferase P1; RARβ2, retinoic acid receptor β2; DAKP, death associated protein kinase; MGMT, 0–6 methylguanine DNA methyltransferase; ER, oestrogen receptor; APC, adenomatous polyposis coli; MSP, methylation specific PCR; ND, not done; HRPCa, hormone refractory prostate cancer.

**Table 2:** DNA methylation as a Prognostic and Predictive Biomarker.

Tumour Type	Marker	Significance	Reference
Oesophageal	APC	Reduced survival (with high levels of methylation)	(Kawakami et al. 2000)
Breast	RASSF1A APC	Independent prognostic markers RR death 5.7	(Muller et al. 2003)
Breast	RASSF1A	Persistence of plasma RASSF1A after 1 year of therapy associated with poor outcome	(Muller et al. 2003)
NSCLC	14-3-3σ	Longer survival in patients treated with cisplatin and gemcitabine chemotherapy	(Ramirez et al. 2005)
NSCLC	APC	High APC methylation associated with poor survival	(Usadel et al. 2002)
Melanoma	RASSF1A RAR β2	Worse overall survival with at least 1 methylated gene. Methylated RASSF1A correlated with biochemotherapy response	(Mori et al. 2005)
Prostate	GSTP1	Predicts for PSA recurrence following prostatectomy HR 4.4	(Bastian et al. 2005)
